# PGC-1 isoforms and their target genes are expressed differently in human skeletal muscle following resistance and endurance exercise

**DOI:** 10.14814/phy2.12563

**Published:** 2015-10-05

**Authors:** Mika Silvennoinen, Juha P Ahtiainen, Juha J Hulmi, Satu Pekkala, Ritva S Taipale, Bradley C Nindl, Tanja Laine, Keijo Häkkinen, Harri Selänne, Heikki Kyröläinen, Heikki Kainulainen

**Affiliations:** 1 Department of Biology of Physical Activity, University of JyväskyläJyväskylä, Finland; 2 Department of Health Sciences, University of JyväskyläJyväskylä, Finland; 3 The Military Performance Division, The Unites States Army Research Institute of Environmental MedicineNatick, Massachusetts; 4 Jyväskylä Central HospitalJyväskylä, Finland; 5 LIKES Research Center for Sport and Health SciencesJyväskylä, Finland

**Keywords:** *PGC1-1β*, *PGC-1α*, physical activity, splice variant

## Abstract

The primary aim of the present study was to investigate the acute gene expression responses of PGC-1 isoforms and PGC-1α target genes related to mitochondrial biogenesis (*cytochrome C*), angiogenesis (*VEGF-A*), and muscle hypertrophy (myostatin), after a resistance or endurance exercise bout. In addition, the study aimed to elucidate whether the expression changes of studied transcripts were linked to phosphorylation of AMPK and MAPK p38. Nineteen physically active men were divided into resistance exercise (RE, *n* = 11) and endurance exercise (EE, *n* = 8) groups. RE group performed leg press exercise (10 × 10 RM, 50 min) and EE walked on a treadmill (∼80% HR_max_, 50 min). Muscle biopsies were obtained from the vastus lateralis muscle before, 30 min, and 180 min after exercise. EE and RE significantly increased the gene expression of alternative promoter originated *PGC-1α* exon 1b- and 1bxs’-derived isoforms, whereas the proximal promoter originated exon 1a-derived transcripts were less inducible and were upregulated only after EE. Truncated *PGC-1α* transcripts were upregulated both after EE and RE. Neither RE nor EE affected the expression of *PGC-1β*. EE upregulated the expression of *cytochrome C* and *VEGF-A*, whereas RE upregulated *VEGF-A* and downregulated *myostatin*. Both EE and RE increased the levels of p-AMPK and *p-*MAPK p38, but these changes were not linked to the gene expression responses of *PGC-1* isoforms. The present study comprehensively assayed *PGC-1* transcripts in human skeletal muscle and showed exercise mode-specific responses thus improving the understanding of early signaling events in exercise-induced muscle adaptations.

## Introduction

Skeletal muscle comprises about 40–50% of body mass in humans (lean) and plays significant roles in locomotion, heat production, and whole-body metabolism. Skeletal muscle has an outstanding capability to adapt to a variety of external stimuli that explains, in part, the marked differences observed in physical performance (e.g., endurance and strength) and health profiles between individuals (Hawley et al. 2014). The question remains, Which specific mechanisms are involved in the response to different types of physical activities? Because the effects are so diverse, mechanisms comprise multiple signaling cascades that form a complex network with each other (Egan et al. 2010). Yet, there have been attempts to identify single signaling cascades or molecules that could work as a master regulator for controlling exercise-specific adaptations (Atherton et al. 2005; Baar and Esser 1999). In recent years, the transcriptional coactivator peroxisome proliferator-activated receptor-*γ* coactivator (PGC)-1*α* has been under a thorough investigation. PGC-1*α* has been identified as a regulator of mitochondrial biogenesis, angiogenesis, antioxidant defense, and inflammatory proteins (Olesen et al. 2010).

A single bout of prolonged endurance exercise transiently increases *PGC-1α* mRNA content in human and rat skeletal muscle (Pilegaard et al. 2003; Gidlund et al. 2015; Baar et al. 2002; Chinsomboon et al. 2009). Furthermore, other types of physical activity (e.g., sprint and resistance exercise) can also result in an increase in the gene expression of *PGC-1α* (Gibala 2009; Ydfors et al. 2013). There are several intracellular signaling pathways that may contribute to eliciting the exercise-induced *PGC-1α* gene expression response including calcium signaling, AMPK and MAPK signaling, ROS-mediated regulation, and *β*-adrenergic signaling (Olesen et al. 2010).

Recently the existence of several different splice variants of *PGC-*1*α* has been found in skeletal muscle. The splice variants differ from their starting exon (exon 1a, exon 1b, and exon 1b’/1c) and via alternative 3′ splicing, which produce either full-length PGC-1α protein (PGC-1α) or shorter N-truncated protein (NT-PGC-1α). Exon 1a-derived *PGC-1α* mRNAs are transcribed from the canonical proximal promoter, while exon 1b- and 1b’-derived mRNAs are transcribed from an alternative promoter ∼14-kb upstream from the canonical one (Ruas et al. 2012; Zhang et al. 2009). Lately, the roles of different splice variants of *PGC-1α* in the initial signaling events of exercise-induced skeletal muscle adaptations have been under heavy investigation. Ruas et al. (2012) showed that the expression of *PGC-1α4*, exon 1b originated N-truncated *PGC-1α* transcript, results in robust skeletal muscle hypertrophy in vitro and in vivo. The study suggested that PGC-1α4 is preferentially induced in mouse and human muscle during resistance exercise, and this would lead to muscle hypertrophy via induced expression of IGF1 and repressed expression of myostatin. However, the studies of Lundberg et al. (2014) and Ydfors et al. (2013) have questioned the preferential induction of *PGC-1α4* expression after resistance exercise by showing that also endurance exercise has effects on the response of this transcript in human skeletal muscle. In fact, the effects of endurance exercise were shown already in the study of Ruas et al. (2012). Interestingly, the study of Thom et al. (2014) showed that hypoxia specifically induces truncated forms of *PGC-1α* (*NT-PGC-1α* and *PGC-1α4*), which induces *VEGF* expression and angiogenesis, while having only a little effect on mitochondrial genes. Previous studies have also shown that both resistance and endurance exercise are able to induce expression of alternative and proximal promoter originated *PGC-1α* transcripts, the alternative promoter originated transcripts being much more inducible compared to proximal promoter originated transcripts (Lundberg et al. 2014; Ydfors et al. 2013).

Exact primer design is an essential part of studies measuring mRNA levels of different splice variants. Since the most of the previous studies (Lundberg et al. 2014; Norrbom et al. 2011; Ydfors et al. 2013) investigating exercise-induced acute gene expression responses of different *PGC-1α* splice variants in human skeletal muscle did not report exact primer sequences, it was impossible to evaluate the specificity of the used primers. To increase knowledge of early signaling events that drive the exercise mode-specific adaptations, and to confirm previous findings with well-defined primers, the present study aimed to investigate the acute gene expression responses of different *PGC-1* isoforms after a single bout of high-load resistance exercise (leg press protocol) or moderate intensity endurance exercise (uphill walking on treadmill) in human skeletal muscle. In addition, we aimed to determine how these two different exercise protocols affect the expression of a selection of known PGC-1α target genes related to mitochondrial biogenesis, angiogenesis, and muscle hypertrophy. Furthermore, we wanted to elucidate whether the expression changes of studied transcripts were linked to phosphorylation changes of known *PGC-1α* regulators AMPK and p38 MAPK.

## Materials and Methods

### Ethical approval

The study was conducted according to the Declaration of Helsinki, and ethical approval was granted by the ethics committees of the University of Jyväskylä and the Central Finland Health Care District, Jyväskylä, Finland. Each subject was carefully informed of all potential risks and discomforts and, thereafter, signed an informed consent document.

### Study subjects

Two different experimental setups were included in the study: resistance and endurance exercise. The studies were linked to the larger military research project. A total of 22 healthy male reservists, who were physically active but not endurance or strength athletes, were recruited for the present investigation. Two subjects withdrew from the endurance exercise group and one subject was excluded from this group because of inadequate muscle samples. Therefore, the final study groups included 11 subjects in resistance exercise group (RE) and eight in endurance exercise group (EE). The general characteristics of the groups, which have been partly published previously (Ahtiainen et al. 2015), were as follows (mean ± SD): RE (*n* = 11, 26.0 ± 4.6 years, 182 ± 8 cm, 78.6 ± 11.7 kg, MVC 339.7 ± 99.7 kg, CMJ 34.0 ± 3.8 cm, 1RM 207.0 ± 26.6 kg, VO_2max_ 59.9 ± 5.3 mL·kg^−1^·min^−1^) and EE (*n* = 8, 27.0 ± 3.6 years, 181 ± 9 cm, 72.5 ± 11.5 kg, MVC 221.8 ± 33.2 kg, CMJ 30.9 ± 3.6 cm, 1RM 167.3 ± 33.6 kg, VO_2max_ 65.3 ± 5.2 mL·kg^−1^·min^−1^).

### Exercise protocols

The protocols and equipment used with both exercise modes have been described in detail elsewhere (Ahtiainen et al. 2015). In brief, RE was a single high-load hypertrophic type of resistance exercise consisting 10 sets of 10 repetition maximum (10 × 10 RM) using a bilateral leg press device (David 210, David Health Solutions Ltd., Helsinki, Finland). The starting load was 70% of 1RM. Thereafter, the loads were adjusted so that each subject would be able to perform a maximum of 10 repetitions for each set. The recovery between the sets was 2 min, except a 10 min rest that was applied between the fifth and sixth set. EE consisted of 50 min of strenuous walking on a treadmill. To increase exercise load additional weight (16.5 kg) were carried in backpack (OJK-1, Telineyhtymä, Kotka, Finland). At minutes 0:00–5:00 and 40:00–45:00, the speed of the treadmill was 4.5 km·h^−1^ and the slope 4.0 degrees. At minutes 5:00–10:00 and 45:00–50:00, the speed was 7.0 km·h^−1^ and the slope 4.0 degrees. During the minutes 10:00–40:00, the walking speed was individually controlled and adjusted in 5-min intervals by heart rate (HR) and blood lactate measurements. The criteria for walking speed included (1) blood lactate concentration approximately 4.0 mmol·L^−1^; and (2) HR between 75% and 85% of the individual HR maximum (HR_max_) that was determined by a maximal endurance capacity test before the study.

The subjects were familiarized with the upcoming exercises and testing protocols 4–5 days before the study. To control effects of nutrition and hydration status, the subjects fasted for 12 h before the first (PRE) biopsy. Immediately after the biopsy, the subjects ate an energy bar (170 kcal, protein 7 g, carbohydrate 21 g, and fat 5.5 g) and drank 0.5 L of water. Two hours after the exercise loading, the subjects were allowed to eat again an energy bar and drink water (ad libitum). The energy bars and water were given to avoid lack of energy and disturbance of fluid balance during the measurement day lasting 8.5 h. Moreover, the amount of given energy was selected so that we could minimize the known effects of caloric restriction, fasting, and feeding on exercise responses (Ranhotra 2010; Canto et al. 2010). The subjects were asked to refrain from vigorous physical activity for 2 days before the study.

### Muscle strength and explosive power

To assess basal strength characteristics and muscular fatigue produced by exercise, the following measurements were performed before and after exercise: maximal voluntary bilateral isometric force (MVC) of leg extensor muscles (electromechanical dynamometer, Department of Biology of Physical Activity, University of Jyväskylä, Jyväskylä, Finland) (Ahtiainen et al. 2015), maximal dynamic bilateral 1RM leg press (David 210, David Health Solutions Ltd, Helsinki, Finland), and countermovement jump (CMJ).

### Blood lactate

In both EE and RE, fingertip blood lactate samples were collected before and immediately after exercises into capillary tubes, which were placed in a 1-mL hemolyzing solution and analyzed automatically (EKF diagnostic, Biosen, Barleben, Germany). To adjust speeds in EE, blood lactate was monitored (Lactate Pro LT-1710 analyser 35, Arkray Inc., Kyoto, Japan) during the exercise in 5-min intervals.

### Muscle biopsy procedure

The first (PRE) muscle biopsy was obtained from VL (right leg) 3 h before exercise. Biopsies were taken with a 5-mm Bergström biopsy needle together with suction, midway between the patella and greater trochanter. Muscle depth was kept constant in different biopsies within subject by markings on the needle. The muscle sample was cleaned of any visible connective and adipose tissue as well as blood and frozen immediately in liquid nitrogen (−180°C) and stored at −80°C. The postexercise biopsy samples were taken from VL (left leg) after 30 and 180 min of recovery. The recovery protocol was identical in both exercise groups. The subjects were physically passive during the recovery.

### Western blot analysis

Muscle biopsy specimens were hand-homogenized in ice-cold buffer (20 mmol·L^−1^ HEPES [pH 7.4], 1 mmol·L^−1^ EDTA, 5 mmol·L^−1^ EGTA, 10 mmol·L^−1^ MgCl_2_, 100 mmol·L^−1^ b-glycerophosphate, 1 mmol·L^−1^ Na_3_PO_4_, 2 mmol·L^−1^ DTT, 1% Triton X-100, 0.2% sodium deoxycholate, 30 *μ*g·mL^−1^ leupeptin, 30 *μ*g·mL^−1^ aprotinin, 60 *μ*g·mL^−1^ PMSF, and 1% phosphatase inhibitor cocktail; P 2850; Sigma, St. Louis, MO) at a dilution of 15 *μ*L·mg^−1^ of wet weight muscle. Homogenates were rotated for 30 min at 4°C, centrifuged at 10,000 *g* for 10 min at 4°C to remove cell debris, and stored at −80°C. Total protein content was determined using the bicinchoninic acid protein assay (Pierce Biotechnology, Rockford, IL).

Aliquots of muscle lysate, containing 30 *μ*g of total protein, were solubilized in Laemmli sample buffer and heated at 95°C for 10 min to denature proteins, and were then separated by SDS-PAGE for 90 min at 200 V using 4–20% gradient gels on Criterion electrophoresis cell (Bio-Rad Laboratories, Hercules, CA). All samples from each subject were run on the same 18-sample gel. Proteins were transferred to PVDF membranes at 350 mA constant current for 3 h on ice at 4°C. Membranes were blocked in TBS with 0.1% Tween 20 (TBS-T) containing 5% nonfat dry milk for 1 h and then incubated overnight at 4°C with rabbit polyclonal primary antibodies. Antibodies recognizing phosphorylated p38MAPK^Thr180/Tyr182^ and AMPK*α*^Thr172^ were purchased from Cell Signaling Technology (Danvers, MA) and these primary antibodies were diluted 1:2000 in TBS-T containing 2.5% nonfat dry milk. For measuring protein levels of nontruncated full-length splice variants of PGC-1*α*, the antibody (1:3000, Calbiochem, Merck KGaA, Darmstadt, Germany) against C-terminus of protein (amino acids 777–797) was used. Membranes were then washed (5 × 5 min) in TBS-T, incubated with secondary antibody (horseradish peroxidase-conjugated anti-rabbit IgG; Cell Signaling Technology) diluted 1:25,000 in TBS-T with 2.5% milk for 1 h followed by washing in TBS-T (5 × 5 min). Proteins were visualized by ECL according to the manufacturer’s protocol (SuperSignal west femto maximum sensitivity substrate, Pierce Biotechnology) and quantified (band intensity × volume) using a ChemiDoc XRS in combination with Quantity One software (version 4.6.3. Bio-Rad Laboratories).

The uniformity of protein loading was confirmed by staining the membrane with Ponceau S. Our earlier studies and preliminary experiments confirmed a proportional linear relation between the protein loaded and the strongest band in Ponceau S at ∼42 kDa in quantification between 5 and 60 *μ*g of total protein loaded (Hulmi et al. 2012). A less well proportional and linear relationship was found between GAPDH, *α*-actin, and staining of myosin heavy chain left in the gel after blotting. Therefore, all of the results were normalized to the corresponding Ponceau S staining value at ∼42 kDa. In addition, to reduce gel-to-gel variation, the results of all samples within the gel were normalized to the mean value of all PRE samples within the gel. Quantification of p-p38 MAPKThr180/Tyr182 was based on the average of two visible bands at 42 and 44 kDa. Phosphorylated AMPKαThr172 was quantified using the visible band at 62 kDa and nontruncated PGC-1*α* using the band at 100 kDa.

### RNA extraction and cDNA synthesis

Total RNA was isolated from the muscle biopsy with Trizol reagent (Life Technologies, Carlsbad, CA) according to the manufacturer’s instructions. Muscle samples were homogenized with a FastPrep FP120 (Thermo Fisher Scientific, Waltham, MA) tissue homogenizer by using Lysing Matrix D FP120 (Thermo Fisher). The quality of RNA was confirmed by spectrophotometry (NanoDrop; Thermo Fisher Scientific) and agarose gel electrophoresis. Reverse transcription of mRNA was performed from total RNA (5 *μ*g) by using anchored oligo(dT)_20_ primers (Oligomer, Helsinki, Finland) and a SuperScript III Reverse Transcriptase kit (Life Technologies) according to the manufacturer’s instructions. The cDNA samples were stored in −20°C.

### Real-time quantitative PCR

*Cytochrome C* (*CYC*), *glyceraldehyde-3-phosphate dehydrogenase* (*GAPDH*), and *vascular endothelial growth factor A* (*VEGF-A*) mRNAs were quantified using TaqMan primers and hydrolysis probes (Assay IDs: *CYC*: Hs01588974_g1; *GAPDH*: Hs03929097_g1; *VEGF-A*: Hs00900055_m1), TaqMan Gene Expression Master Mix (Life Technologies), and ABI 7300 Real-Time quantitative PCR System (Life Technologies). The PCR cycle parameters used were: +50°C for 2 min, +95°C for 10 min, 40 cycles at +95°C for 15 sec, and +60°C for 1 min. The other studied transcripts (*PGC-1α* exon 1a-, 1b- and 1b’ -derived mRNAs, total *NT-PGC-1α*, total *PGC-1α*, *PGC-1β*, and *myostatin*) were quantified using iQ SYBR Supermix (Bio-Rad Laboratories) and CFX96 Real-Time PCR Detection System (Bio-Rad Laboratories). The PCR cycle parameters used with this system were as follows: +95°C for 10 min, 40 cycles at +95°C for 10 sec, at gene-specific annealing temperature (61°C, except 56 for total *NT-PGC-1α*) for 30 sec and at +72°C for 30 sec, followed by 5 sec at +65°C.

**Figure 1:**
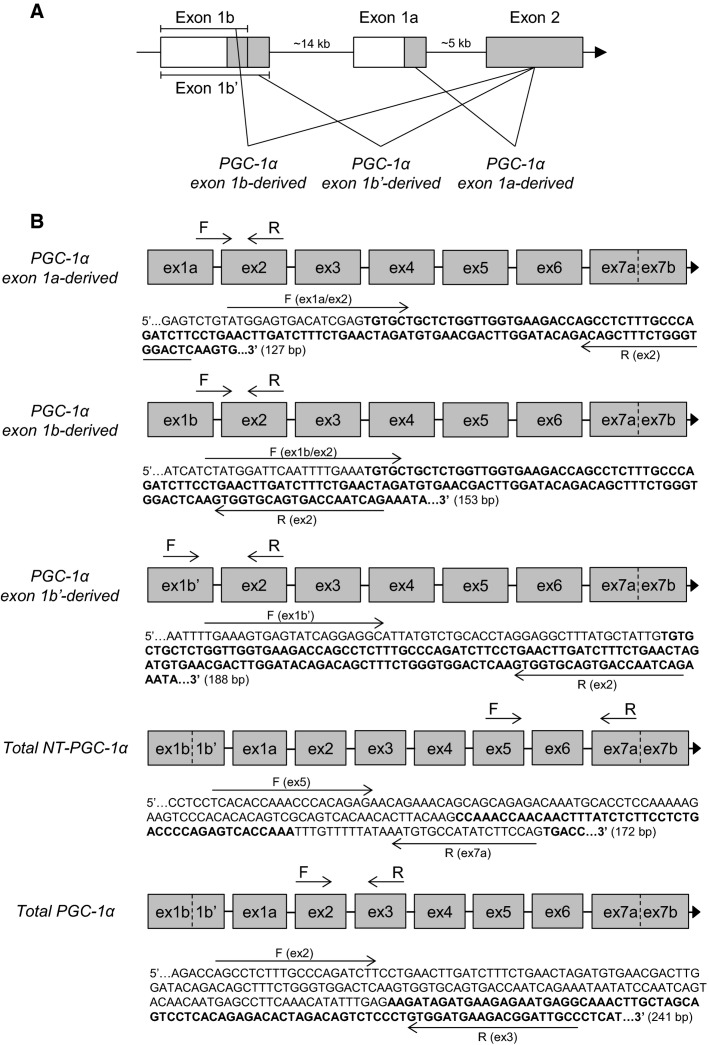
(A) The schematic structure of the 5′ region of the human *PGC-1α* gene (Miura et al. 2008). White boxes indicate untranslated exon regions and gray boxes translated coding regions. Straight lines between boxes indicate introns. When mRNA is formed from the gene, two completely distinct first exons of *PGC-1α* (exon 1a and 1b) can be spliced to the common exon 2. Furthermore, exon 1b can be spliced to the common exon 2 in two different ways producing either *PGC-1α* exon 1b- or 1b’-derived transcripts. In addition to differences in the starting exon, the splice variants of *PGC-1α* differ via alternative 3′ splicing. The exon subsequent to exon 6 may be either exon 7a (ex7a) or exon 7b (ex7b). Ex7a is the exon insert, which contains an in-frame stop codon resulting in the shorter N-truncated proteins (*NT-PGC-1α*). Ex7b is present in nontruncated full-length *PGC-1α* proteins, which are translated using all 13 exons of *PGC-1α*. The proximal promoter drives the transcription of *PGC-1α* exon 1a-derived transcripts (truncated and nontruncated) and the alternative promoter the transcription of *PGC-1α* exon 1b and 1b’-derived transcripts (truncated and nontruncated). (B) Detailed descriptions of primer pairs and their possible cDNA targets are presented. Primer pairs for detecting *PGC-1α* exon 1a-, 1b-, 1b’-derived transcripts are specific for different first exons, but do not separate truncated and nontruncated *PGC-1α* splice variants. Primer pair for total *NT-PGC-1α* is specific for truncated *PGC-1α* splice variants, but do not separate differences in first exon. The total *PGC-1α* primer pair was designed to detect all truncated and nontruncated splice variants of *PGC-1α*. The possible exon structures of mRNA targets for each primer pair are illustrated with gray boxes. The structure is presented only from the first exon to Ex7a or Ex7b because the mRNA structure is common in following exons (Ex8–Ex13). Binding sites of target specific forward (F) and reverse (R) primers are pointed out by arrows drawn next to the cDNA sequence in question. The sequences of all primers are also listed in Table1. The sequences are amplicon sequences plus five nucleotides in the 3′ and 5′ end. The sequences of every other exon are bolded to separate subsequent exons from each other. The sequences were constructed by using human PGC-1*α* cDNA sequence (Ensembl: ENST00000264867), human PGC-1*α* gene sequence with its upstream regions (NCBI, Gene ID: 10891), and published cDNA structures of murine PGC-1*α* splice variants (Wen et al. 2014; Ruas et al. 2012).

**Figure 2:**
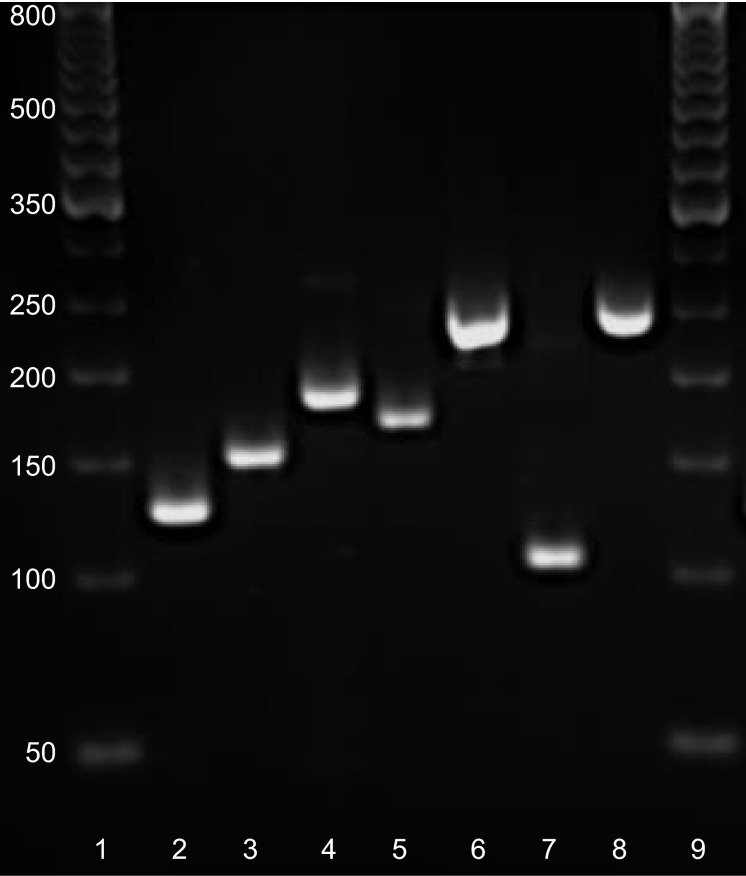
RT-qPCR products on agarose gel. The bands correspond to the calculated amplicon sizes: *PGC-1α* exon 1a- (127 bp, lane 2), 1b- (153 bp, lane 3), 1b’ (188 bp, lane 4)-derived transcripts, total *NT-PGC-1α* (172 bp, lane 5), total *PGC-1α* (241 bp, lane 6), *PGC-1β* (104 bp, lane 7), and *myostatin* (243 bp, lane 8). 50-bp DNA ladder at lanes 1 and 9.

The primer sequences recognizing total *NT-PGC-1α* and *PGC-1α* exon 1a-derived transcripts were copied from Ruas et al. (2012) and *myostatin* from Kim et al. (2007). Other primers were designed with Primer3 (web version 4.0.0) software (Koressaar and Remm 2007; Untergasser et al. 2012). The structure of 5′ region of the human *PGC-1α* gene is illustrated in Figure1A. In addition, the detailed description of exon structure and primer design of measured *PGC-1α* transcripts is presented in Figure1B. The sequences of primer sets used with the SYBR green method are listed in Table1. Following pilot RT-qPCR runs performed with the SYBR green method, the specificity of each primer set was monitored by the melting curves and by agarose gel (3%) electrophoresis (Fig.2). In addition, optimal gene-specific annealing temperatures were determined. The amplification efficiencies for each gene were between 95% and 105%.

**Table 1:** RT-qPCR primers used with the SYBR green method

Transcript	Strand	Sequence 5′–3′
*PGC-1α* exon 1a derived	Forward	ATGGAGTGACATCGAGTGTGCT
	Reverse	GAGTCCACCCAGAAAGCTGT
*PGC-1α* exon 1b derived	Forward	CTATGGATTCAATTTTGAAATGTGC
	Reverse	CTGATTGGTCACTGCACCAC
*PGC-1α* exon 1b’ derived	Forward	TGAAAGTGAGTATCAGGAGGCA
	Reverse	CTGATTGGTCACTGCACCAC
Total *NT-PGC-1α*	Forward	TCACACCAAACCCACAGAGA
	Reverse	CTGGAAGATATGGCACAT
Total *PGC-1α*	Forward	AGCCTCTTTGCCCAGATCTT
	Reverse	GGCAATCCGTCTTCATCCAC
*PGC-1β*	Forward	GAGTCAAAGTCGCTGGCATC
	Reverse	AACTATCTCGCTGACACGCA
*Myostatin*	Forward	CTACAACGGAAACAATCATTACCA
	Reverse	GTTTCAGAGATCGGATTCCAGTAT

**Figure 3:**
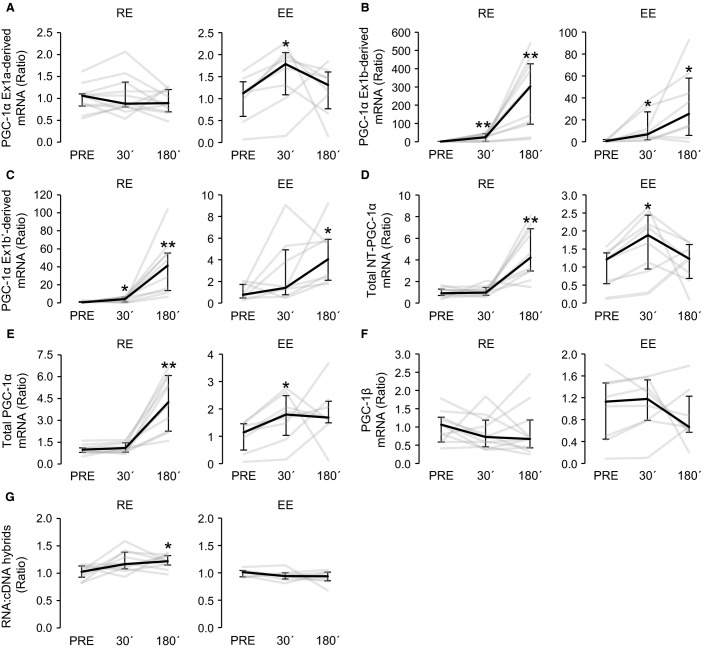
The expression profiles of *PGC-1α* exon 1a-derived transcripts (A), *PGC-1α* exon 1b-derived transcripts (B), *PGC-1α* exon 1b’-derived transcripts (C), total *NT-PGC-1α* (D), total *PGC-1α* (E), and *PGC1β* (F) at 30 min (30′) and 180 min (180′) after resistance (RE, *n* = 11) and endurance (EE, *n* = 8) exercises. The gene expression data of each sample are normalized to corresponding relative concentration value of RNA:cDNA hybrids (G). The gene expression changes are presented in relation to PRE average. Black lines represent median responses to exercise and gray lines represent individual responses. Error bars represent interquartile range. *Wilcoxon matched pairs signed-rank test with Holm–Bonferroni correction *P *<* *0.05 versus PRE, **P <* *0.01 versus PRE.

Each sample was analyzed in duplicate, and a nontemplate control was included in each run. Relative gene expression levels of all target transcripts were normalized using RNA:cDNA hybrid concentrations which were measured using a Quant-iT™ PicoGreen® assay (Life Technologies) according to the manufacturer’s recommendations. This method of normalization has been validated especially for human exercise studies and muscle biopsy samples (Lundby et al. 2005). The stability of RNA:cDNA hybrid concentrations was compared to stability of *GAPDH*, one of the most common reference genes used in exercise studies. The average differences between time points in both exercise modes were similar in *GAPDH* and RNA:cDNA hybrid concentrations. However, the variation within time points in both exercise modes were lower in RNA:cDNA hybrid concentrations compared to *GAPDH* levels. The RNA:cDNA hybrid concentrations are presented in (Fig.3G). The normalized relative gene expression results (R) of each sample were expressed in relation to PRE average and calculated using following formula:

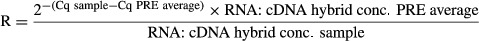


### Statistical analyses

Conventional statistical methods were used to obtain means, standard deviations (SD), medians, interquartile ranges (IQR), and percentiles. The Shapiro–Wilk test was used to test the normality of the variables and the Levene’s test was used to analyze the homogeneity of variances. Due to the small sample size and random violations in the normal distribution assumption and the homogeneity of the variance assumption, a nonparametric Friedman’s two-way ANOVA by ranks test was used to determine differences between time points (PRE, 30′ and 180′) within groups (RE and EE). Wilcoxon matched-pair signed-rank test with Holm–Bonferroni correction was used for the post hoc analysis. A Spearman’s rank correlation test was used to test if the changes in p38 MAPKThr180/Tyr182 or AMPKαThr172 phosphorylation were associated with exercise-induced gene expression changes. The significance level was set at *P *<* *0.05. All statistical analyses were carried out using IBM SPSS statistics 20 software (IBM Corporation, Armonk, NY).

## Results

### The effects of exercise bouts

RE led to a decrease of 45 ± 16% (*P *<* *0.01) in MVC and 17 ± 24% (NS, *P *=* *0.07) in CMJ from pre- to postexercise. Blood lactate increased to 11.1 ± 3.0 mmol·L^−1^ (*P *<* *0.01) immediately after RE. During the loading in EE, averaged treadmill speed was 6.2 ± 0.4 km·h^−1^ and the slope 4.1 ± 0.8 degrees, the blood lactate was 4.2 ± 1.0 mmol·L^−1^ and HR was 83 ± 8% of the HR_max_. Immediately after EE, MVC was 9 ± 14% (NS, *P *=* *0.06) and CMJ 7 ± 6% (*P *<* *0.05) lower than before the exercise, and blood lactate was 2.4 ± 1.5 mmol·L^−1^ (*P *<* *0.01). These results (except CMJ) have been previously published (Ahtiainen et al. 2015).

### Gene expression responses of *PGC-1* transcript variants after RE and EE

The average basal state (PRE) C_q_ values (all subjects) for studied *PGC-1* transcripts were as follows: *PGC-1α* exon 1a-derived transcripts 25.6 ± 1.2, *PGC-1α* exon 1b-derived transcripts 31.3 ± 3.1, *PGC-1α* exon 1b’-derived transcripts 33.8 ± 1.7, total *NT-PGC-1α* 29.7 ± 1.4, total *PGC1α* 24.1 ± 1.2, and *PGC1β* 30.0 ± 1.6. The expression of *PGC-1α* exon 1a-derived transcripts increased by 1.7-fold (*P *<* *0.05, Fig.3A) 30 min after EE. After RE no significant response was detected. The expression of *PGC-1α* exon 1b-derived transcripts was significantly increased in response to both RE and EE (Fig.3B), while the peak changes were detected 180 min after exercise. At this time point, the average gene expression change was 170-fold (*P *<* *0.05) after EE and 997-fold (*P *<* *0.01) after RE. The expression of *PGC-1α* exon 1b’-derived transcripts were low in the present basal conditions. In the RE group, the transcripts were detected in six PRE samples from 11 and in EE group in three PRE samples from eight. However, after RE and EE the expression of *PGC-1α* exon 1b’-derived transcripts was substantially increased (Fig.3C). When PRE values below the detection limit (*C*_q_ value before of which amplification was repeatable and specific) were replaced with the detection limit values (*C*_q_ 34.9), the average change was 67-fold at 180 min after RE (*P *<* *0.01) and ninefold at 180 min after EE (*P *<* *0.05). The expression of truncated *PGC-1α* transcripts (total *NT-PGC-1α*) (Fig.3D) was increased at 180 min after RE (fivefold, *P *<* *0.01) and 30 min after EE (1.7-fold, *P *<* *0.01). The expression of total *PGC1α* (all truncated and nontruncated splice variants together) (Fig.3E) was increased at 180 min after RE (fourfold, *P *<* *0.01) and 30 min after EE (1.8-fold, *P *<* *0.05). *PGC1β* had no significant gene expression response to either of the exercises (Fig.3F).

### Gene expression responses of PGC-1*α*-regulated genes after RE and EE

**Figure 4:**
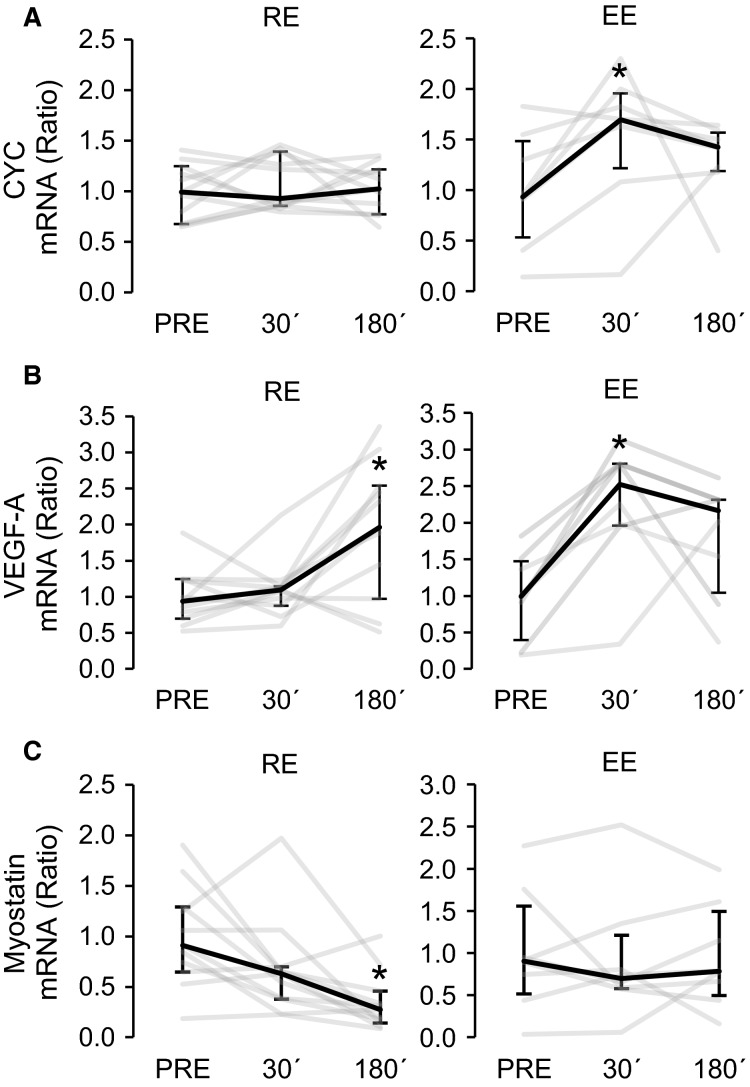
Gene expression changes of known PGC-1*α* target genes *cytochrome C* [(A), *CYC*], *VEGF-A* (B), and *myostatin* (C) at 30 min (30′) and 180 min (180′) after resistance (RE, *n* = 11) and endurance (EE, *n* = 8) exercises. The gene expression changes are presented in relation to PRE average. Black lines represent median responses to exercise and gray lines individual responses. Error bars represent interquartile range. *Wilcoxon matched pairs signed-rank test with Holm–Bonferroni correction *P *<* *0.05 versus PRE, **P <* *0.01 versus PRE.

The gene expression of mitochondrial marker *cytochrome c* was increased at 30 min after EE (1.7-fold, *P *<* *0.05, Fig.4A), but there were no responses to RE. However, the gene expression of angiogenesis regulator *VEGF-A* was increased both after EE (threefold, *P *<* *0.05) and RE (twofold, *P *<* *0.05). EE-induced response was significant already 30 min after exercise, but RE-induced response did not appear until 180 min after exercise (Fig.4B). RE decreased (2.5-fold, *P *<* *0.05) the gene expression of *myostatin*, the known inhibitor of muscle growth, without response to EE (Fig.4C).

### The level of PGC-1*α* proteins, p-p38 MAPK^Thr180/Tyr182^ and p*-*AMPKα^Thr172^, and their associations with studied gene expression changes

**Figure 5:**
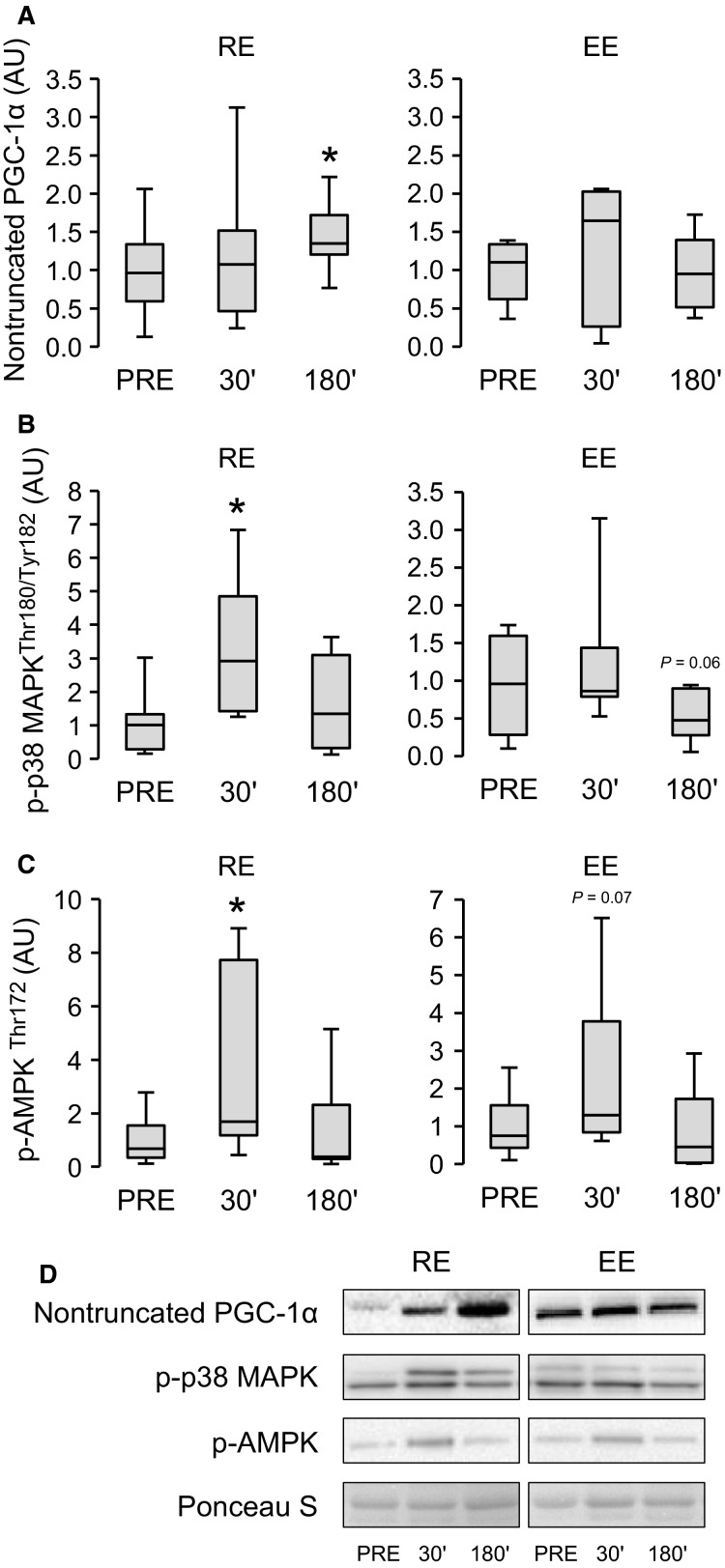
The effect of endurance (EE, *n* = 7) and resistance (RE, *n* = 9) exercises on levels of nontruncated PGC-1*α* proteins (A), p-p38-MAPK (B), and p-AMPK (C) at 30 min (30′) and 180 min (180′) after exercise. The results are presented in relation to PRE average and the box plots show minimum–maximum values, interquartile ranges, and medians. *Wilcoxon matched pairs signed-rank test with Holm–Bonferroni correction *P *<* *0.05 versus PRE. Representative immunoblot images of one individual (D).

The protein level of full-length nontruncated PGC-1*α* was increased at 180 min after RE (2.3-fold, *P *<* *0.05, Fig.5A) without changes after EE (*n* = 7). The results of phosphorylated p38 MAPK^Thr180/Tyr182^ and AMPKα^Thr172^ have been previously published from pre to post 30 min (Ahtiainen et al. 2015). The present study adds to that study a new time point (180 min) and because of this, the data were reanalyzed using different statistics, and the gel-to-gel variation was normalized differently. The level of the active phosphorylated form of p38 MAPK^Thr180/Tyr182^ was increased by ninefold (*P *<* *0.05, Fig.5B) at 30 min after RE, but not after 180 min or EE. The level of p-AMPKα^Thr172^ was increased by fourfold at 30 min after RE (Fig.5C), but not anymore after 180 min. There was also a trend (*P *=* *0.07) for increased levels of p-AMPKα^Thr172^ at 30 min after EE.

There were no strong associations between the exercise-induced changes in p-p38 MAPK^Thr180/Tyr182^ or p-AMPKα^Thr172^ levels and the gene expression responses of *PGC-1* isoforms, *PGC-1β*, and *myostatin* in either of the exercise groups. However, the level change of p-*AMPKα*^Thr172^ (ratio 30′/PRE) associated with the corresponding changes in *VEGF-A* (*r*_s_ = 0.821, *P = *0.023) and *CYC* (*r*_s_ = 0.75, *P = *0.052) at 30 min after EE. Similar associations were also found after EE when the both time points after exercise were pooled (ratio 30′/PRE and ratio 180′/PRE) and analyzed together (*VEGF-A*: *r*_s_ = 0.608, *P = *0.036; *CYC*: *r*_s_ = 0.601, *P = *0.039).

## Discussion

The main findings of this study were that (1) both EE and RE induced significant increase in the gene expression of alternative promoter originated *PGC-1α* exon 1b- and 1b’-derived isoforms, whereas the proximal promoter originated *PGC-1α* exon 1a-derived transcripts were less inducible and were upregulated only after EE; (2) truncated *PGC-1α* transcripts were upregulated markedly after RE and slightly after EE; (3) based on the marker gene expression changes, EE induced responses typical for angiogenesis and mitochondrial biogenesis, while RE induced responses typical for angiogenesis and muscle hypertrophy; (4) both EE and RE increased the levels of phosphorylated p38 MAPK and AMPKα, but these changes were not linked to the gene expression responses of *PGC-1* isoforms.

The primary aim of the present study was to investigate the acute mRNA responses of different *PGC-1* isoforms after high-load resistance exercise and moderate intensity endurance exercise. The RE and EE protocols used in the present study differed significantly from protocols used in other human studies with a similar aim. The RE protocol was designed to represent common hypertrophic RE for leg muscles. It consisted of significantly more sets (10 vs. 4) and had shorter recovery periods (2 vs. 5 min) between sets compared to study of Ydfors et al. (2013) who also used leg press loading in their study. Other studies (Lundberg et al. 2014; Norrbom et al. 2011) have used knee extension ergometers in the RE loading. The present EE protocol consisted of strenuous walking on treadmill. Even though being common recreational exercise mode, the effects of walking exercises on expression of *PGC-1* isoforms have not been studied earlier. All previous studies (Popov et al. 2014; Ydfors et al. 2013; Gidlund et al. 2015) investigating *PGC-1* splice variant responses have used a bicycle loading for EE. The tests of maximal strength (MVC), explosive strength (CMJ), and lactate levels indicated that RE was anaerobic exercise that predictably induced significant fatigue. As intended, the intensity of EE was close to the onset of blood lactate accumulation (4.0 mmol·L^−1^) and EE induced lesser muscular fatigue in leg extensors compared to RE.

The current results together with previous studies (Lundberg et al. 2014; Ydfors et al. 2013; Norrbom et al. 2011; Gidlund et al. 2015) showed clearly that the expression of alternative promoter driven *PGC-1α* transcripts (exon 1b and 1b’-derived) are strongly induced after different types of RE and EE, whereas transcripts originating from proximal promoter (*PGC-1α* exon 1a-derived) are much less inducible. In the present study, proximal promoter driven transcripts were induced only after EE and the magnitude of the response was slight compared to strong responses seen in alternative promoter-driven transcripts after EE (Fig.4A–C). Our study is the first that reports the RE-induced responses of *PGC-1α* exon 1b’-derived transcripts in human skeletal muscle. Popov et al. (2014) have reported earlier the responses of these transcripts after EE, but the reverse primer used in the detection of these transcripts contained two mismatch nucleotides, which may have impaired the specificity and function of this primer pair. The same primer pair with mismatch nucleotides was used earlier also in the study of Ruas et al. (2012) for detection of *PGC-1α3*. The rodent studies have shown that alternative promoter originated *PGC-1α* isoforms, which were also induced in the present study, promote angiogenesis (Chinsomboon et al. 2009), mitochondrial biogenesis, and improve fatty acid oxidation capacity in skeletal muscles (Miura et al. 2008). Despite different exercise modes and protocols, both RE and EE increased the expression of truncated *PGC-1α* transcripts (total *NT-PGC-1α*) similarly to earlier studies (Gidlund et al. 2015; Popov et al. 2014; Ydfors et al. 2013). Interestingly, it has been shown recently that hypoxia specifically induces truncated forms of *PGC-1α* (*NT-PGC-1α* and *PGC-1α4*), which induces *VEGF* expression and angiogenesis, while having only a little effect on mitochondrial genes (Thom et al. 2014). In this study, neither RE nor EE modulated the expression of *PGC-1β* thus supporting the assumption that PGC-1β is not transcriptionally regulated after exercise (Meirhaeghe et al. 2003).

According the conventional dogma, chronic EE (low-intensity to high-volume loading) favors skeletal muscle adaptations (e.g., mitochondrial biogenesis, angiogenesis and improved β-oxidation) enhancing oxidative capacity with modest effect on muscle size (Holloszy and Booth 1976). Conversely, RE (high–intensity to low-volume loading) increases protein accretion promoting muscle hypertrophy and force (Hawley et al. 2014). The present increased gene expression of mitochondrial marker *cytochrome c* by EE but not RE suggests that mitochondrial adaptation was induced preferentially by EE. In contrast, angiogenic response seemed to be stimulated by both EE and RE because both exercise types increased the mRNA expression of angiogenesis regulator *VEGF-A*. As expected, RE decreased the gene expression of *myostatin*, the known target of PGC-1*α*4 (Ruas et al. 2012) and an inhibitor of muscle growth, but the EE did not have any effect. The downregulation of *myostatin* may be a part of hypertrophic response induced by RE (Hulmi et al. 2007). Yet, the importance of the role of myostatin in hypertrophic response to RE in humans is still unclear and needs further experimental evidence.

To check whether the activation changes of known signaling molecules regulating *PGC-1α* expression would explain the expression changes of specific isoforms of *PGC-1*, the levels of p-p38 MAPKThr180/Tyr182 and p-AMPKαThr172 were measured. The levels of p-p38 MAPK were increased after RE and levels of p-AMPKα after both RE and EE. However, the phosphorylation changes of these signaling molecules were not associated with the gene expression responses of *PGC-1* isoforms. This result is not fully consistent with the study of Norrbom et al. (2011), which indicated that AMPK is a major regulator of *PGC-1α* transcripts from the proximal promoter, but the expression of transcripts from an alternative promoter are regulated by *β*-adrenergic signaling in combination with AMPK. Even if AMPK activation did not explain *PGC-1* isoform responses, we found that *VEGF-A* and *CYC* responses 30 min after EE were associated with a corresponding change in the level of p-AMPKα suggesting that AMPK activation may partly explain *VEGF-A* and *CYC* responses seen after EE.

There were some limitations in our study. Because two separate exercise groups were used instead of a crossover design, the experimental setup did not allow direct comparison of gene expression responses between RE and EE. This study included walking endurance exercise, and it should be noted that another type of endurance exercise than continuous strenuous walking might induce different kind of cellular signaling responses in the front thigh muscles. It is also acknowledged that the time point: immediately after exercise could have been more optimal for detecting peak AMPK and p38 MAPK phosphorylation responses. However, the phosphorylation of these signaling proteins may not drop dramatically during the first 30 min after exercise (Bartlett et al. 2012; Sriwijitkamol et al. 2007). The present and previous studies (e.g., Bartlett et al. 2012; Lundberg et al. 2014; Popov et al. 2014; Gidlund et al. 2015) have shown that the studied transcripts seem to peak during the first 3 h after exercise. Thus, the selected time points can be considered justified and valid for the gene expression response detection of the studied genes. Our setup measured only acute exercise responses without physiological outcome variables of long-term training effects, which could be connected to measured acute exercise responses.

In conclusion, this study comprehensively assayed *PGC-1* transcripts in human skeletal muscle and showed specific response profiles after the present RE and EE. The study established that both alternative promoter originated *PGC-1α* exon 1b- and 1b’-derived transcripts are strongly induced after EE and RE, whereas the proximal promoter originated *PGC-1α* exon 1a-derived transcripts are less inducible and were upregulated only after EE. Truncated *PGC-1α* transcripts were upregulated both after EE and RE, thus there was no clear exercise-type specificity observed in the responses of these transcripts. The expression changes of marker genes suggested that EE induced responses typical for angiogenesis and mitochondrial biogenesis, while RE induced responses typical for angiogenesis and muscle hypertrophy. Our results improve the understanding of exercise-type specific early signaling events and support the idea that gene expression responses of *PGC-1α* isoforms may have an important role in exercise-induced muscle adaptations. However, future studies are still required to verify the roles of different *PGC-1α* isoforms in the mechanisms of muscle adaptation.
